# Establishing a Pediatric Hematology-Oncology Program in Botswana

**DOI:** 10.1200/JGO.17.00095

**Published:** 2018-05-14

**Authors:** Jeremy S. Slone, Amanda K. Slone, Oaitse Wally, Pearl Semetsa, Mpho Raletshegwana, Susan Alisanski, Lisa M. Force, Kamusisi Chinyundo, Judith Margolin, Anurag K. Agrawal, Alan R. Anderson, Michael E. Scheurer, Parth S. Mehta

**Affiliations:** **Jeremy S. Slone**, **Amanda K. Slone**, **Kamusisi Chinyundo**, **Judith Margolin**, **Alan R. Anderson**, **Michael E. Scheurer**, and **Parth S. Mehta**, Texas Children’s Cancer and Hematology Centers; **Jeremy S. Slone**, **Amanda K. Slone**, **Susan Alisanski**, **Lisa M. Force**, **Kamusisi Chinyundo**, **Judith Margolin**, **Anurag K. Agrawal**, **Alan R. Anderson**, **Michael E. Scheurer**, and **Parth S. Mehta**, Baylor College of Medicine, Houston, TX; **Oaitse Wally**, University of Botswana; **Oaitse Wally**, **Pearl Semetsa**, and **Mpho Raletshegwana**, Princess Marina Hospital, Gaborone, Botswana; and **Anurag K. Agrawal**, Children’s Hospital and Research Center, Oakland, CA.

## Abstract

**Purpose:**

Annually, 300,000 children are diagnosed with cancer, and the majority of these children live in low- and middle-income countries (LMICs). Currently, there is incomplete information on pediatric cancer incidence, diagnosis distribution, and treatment outcomes in Africa. Since 2007, a pediatric hematology-oncology program has been operating in Botswana through a partnership between the Botswana government, Baylor College of Medicine, and Texas Children’s Hospital.

**Methods:**

To better understand patient characteristics and outcomes at Botswana’s only pediatric cancer program, a hospital-based data base—the Botswana Pediatric Oncology Database—was established in 2014. Children younger than 18 years of age at the time of diagnosis who presented between 2008 and 2015 were included. Data for this study were extracted in February 2016.

**Results:**

Of the 240 potential enrollees, 185 (77%) children met eligibility for this study. The median age was 6.4 years, and 50.8% were male. Leukemia was the most common malignancy representing 18.9% of the cohort and 88.1% of the total cohort had a histopathologic diagnosis. HIV seropositivity was confirmed in 13.5%. The 2-year overall survival of all pediatric cancer diagnoses was 52.4%. Abandonment of treatment occurred in 3.8% of patients.

**Conclusion:**

In the first 9 years of the program, capacity has been developed through a longstanding partnership between Botswana and Baylor College of Medicine/Texas Children’s Hospital that has led to children receiving care for cancer and blood disorders. Although continued improvements are necessary, outcomes to date indicate that children with cancer in Botswana can be successfully diagnosed and treated.

## INTRODUCTION

In 2016, the International Agency for Research on Cancer reported that, annually, 300,000 children younger than 19 years of age are diagnosed with cancer worldwide and an estimated 80,000 children die as a result of cancer each year.^1^

Low- and middle-income countries (LMICs) in Africa experience a high burden of infectious diseases, including HIV/AIDS. Botswana has one of the highest HIV burdens in the world; prevalence was 23% in 2012, but the incidence of HIV has declined during the past decade, mainly as a result of a strong national commitment to addressing HIV.^2^ Partnership between the Republic of Botswana Ministry of Health and Wellness (MOHW), Baylor College of Medicine (BCM), the Baylor College of Medicine International Pediatric AIDS Initiative at Texas Children's Hospital, and Texas Children’s Hospital (TCH) has been pivotal in addressing pediatric HIV/AIDS in Botswana since 1999.^3^ This partnership led to the establishment of the Botswana-Baylor Children’s Clinical Centre of Excellence in 2003, which expanded to include a pediatric hematology-oncology (PHO) program, with the aid of the Texas Children’s Cancer and Hematology Centers (TXCH), at Princess Marina Hospital (PMH) in 2007.^4^ A hospital-based pediatric cancer data base was established at PMH to evaluate the care and outcomes of children with cancer. Here, we report the initial experience of establishing Botswana’s only pediatric hematology-oncology program.

## METHODS

### Study Setting

**Fig 1:**
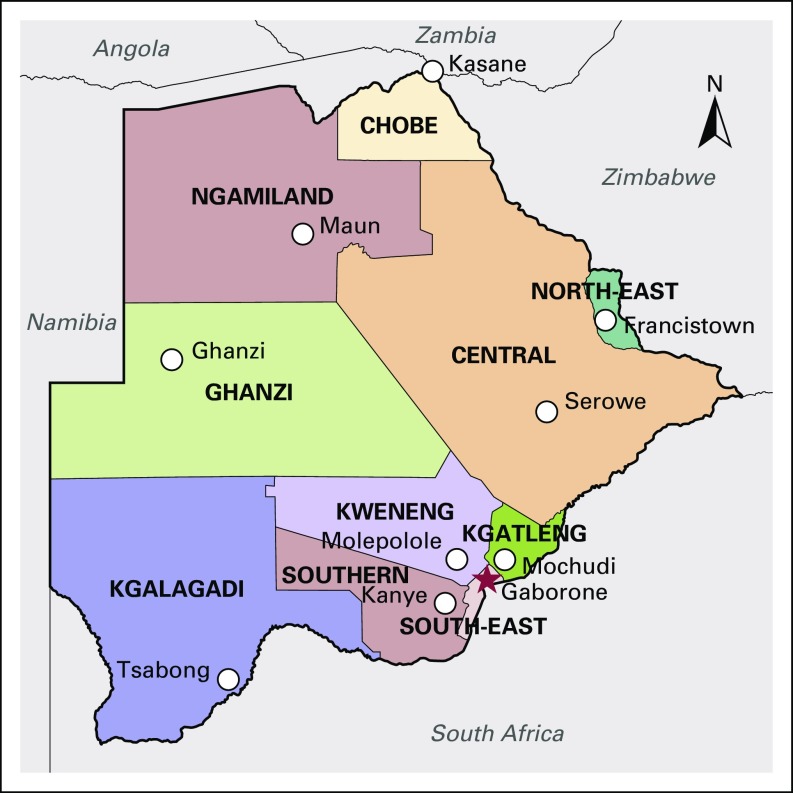
Map of Botswana.

Botswana is a landlocked, upper-middle-income country in Southern Africa roughly the geographic size of Texas (Fig 1).^5,6^ Of the 2.2 million citizens of Botswana, approximately 33% are younger than 14 years of age, and 11,000 children currently live with HIV.^2,6^ PMH, located in the capital of Gaborone, is the main government referral hospital and the only comprehensive cancer and blood disorders center for children in Botswana.

### PHO Services in Botswana

Prior to the BCM/TCH partnership in Botswana, children with cancer in Botswana had little access to specialty services, and there were no pediatric hematologist-oncologists. Only 22 cases of pediatric cancer had been registered at PMH from 2000 to 2007. Since 2007, BCM/TXCH has provided a full-time pediatric hematologist-oncologist to direct the PHO program. Other resources for the PHO program, including nurses, ancillary staff, medications, chemotherapy, and diagnostic studies, are provided through the MOHW and PMH at minimal to no cost to Botswana citizens via the national health care plan. There are no dedicated pediatric oncology nurses, and the pediatric medical ward does not consistently meet published baseline standards for PHO nursing in LMICs.^7,8^

Services available at PMH include hematology and chemistry laboratory, hematopathology, radiology, social services, nutrition services, physiotherapy, pharmacy, pediatric surgery, and other surgical subspecialties. Historically, chemotherapy was prepared by the pediatric hematologist-oncologist before the recent expansion of chemotherapy pharmacy services at PMH. Pathology services are available through the National Health Laboratory, adjacent to PMH, which provides pathology reports on the basis of hematoxylin and eosin staining and, more recently, limited immunohistochemistry. Radiology services include x-ray, ultrasound and computed tomography (CT). Radiation oncology, magnetic resonance imaging (MRI), and additional laboratory tests are available at nearby private facilities through a guarantee of payment from the MOHW and/or private medical aid. Despite the availability of these diagnostic modalities, diagnostic limitations at PMH do not allow complete staging of solid tumors or lymphoma, which likely affects metastatic disease confirmation.

For PHO services unavailable in Botswana, the MOHW can be petitioned to provide funding for these services in the Republic of South Africa. Examples include specialized surgery, such as with CNS tumors, for which surgical resection is feasible and improves prognosis, or implantable central venous access catheters. Because of the unavailability of asparaginase, limited and inconsistent blood component availability, and diagnostic challenges (including access to flow cytometry and cytogenetics), all children with leukemia are referred to the Republic of South Africa for diagnosis and to initiate therapy. Children with acute myelogenous leukemia complete treatment in the Republic of South Africa and children with acute lymphoblastic leukemia (ALL) return to PMH for maintenance therapy with a shared care model with the South African centers. Although most chemotherapy medications are available in Botswana, drug shortages, with both chemotherapy and supportive medications, do occur. For malignancies with a poor prognosis, such as metastatic osteosarcoma, curative therapy is not currently available at PMH, and these patients are treated with a palliative care model.

### Study Participants

To better understand the effect of the pediatric oncology program at PMH and to improve outcomes of pediatric cancer locally, a hospital-based data base, the Botswana Pediatric Oncology Database (BPOD), was established. For this study, inclusion was limited to children with a malignancy diagnosed between January 1, 2008, and December 31, 2015, and age younger than 18 years at the time of diagnosis. Data were extracted in February 2016.

### Data Collection and Statistical Analysis

In 2011, a spreadsheet was started to gather data of all patients referred to the program with suspected cancer and was updated to include patients referred before 2011. It is updated continually to aid clinical management among providers and served as the source for potential BPOD enrollees. Patient records, including but not limited to PMH hospital records, diagnostic reports (laboratory, radiology, pathology), and treatment roadmaps, are maintained by the clinical team. Data were collected from clinical records by the PHO team by using a data collection form and then were entered into the password-protected BPOD. Starting in 2014, retrospective data from the program’s inception was added with ongoing enrollment and routine updates.

Histopathologic diagnosis was defined as a tissue confirmation of the malignancy at any point, including at diagnosis, at delayed primary tumor removal, or on post-mortem examination. HIV testing is a requirement at admission to PMH, so a missed HIV diagnosis is unlikely. We grouped patients with a confirmed HIV-negative status with those who had HIV-unconfirmed status (documentation unavailable) and compared this group with patients who had confirmed HIV-positive status.

Outcomes were determined on the basis of routine clinical follow-up and clinical records. Abandonment of treatment was defined as a greater than 4-week absence from scheduled curative therapy.^9^ For analysis of overall survival (OS), patients were classified as alive if their most recent status was either active on treatment (curative intent) or off treatment versus a final outcome of death, abandonment of treatment, or palliative care. Abandonment of treatment was registered as an event for any child that met the definition, but their final outcome was updated as indicated if they returned to care. Follow-up time was calculated from the date of diagnosis until the first of any of the following: death, palliative care status, abandonment (if did not return to care), or their most recent clinical encounter.

Categoric variables were described as percentages, and continuous variables were described as medians with interquartile ranges. Fisher’s exact test was used to evaluate statistical significance of associations between categoric variables. The Student *t* test was used to compare means for continuous variables. Kaplan-Meier survival analysis was used to evaluate outcomes. Statistical analyses were done using STATA, version 11 (STATA, College Station, TX). The study was approved by the ethics committees of the University of Botswana, BCM, and PMH and by the MOHW Health Research and Development Division.

## RESULTS

Since the inception of the PHO program at PMH in 2007, 349 patients had recorded referrals to the pediatric oncology program at PMH for suspected cancer. Ineligible patients were excluded on the basis of date of diagnosis (n = 36), age at diagnosis (n = 4), or lack of a diagnosis of cancer (n = 69). Of the 240 potentially eligible patients, additional exclusions were performed because of either a lack of information about the diagnosis or outcome (or if their treatment was given outside of PMH; n = 42) or if pending enrollment into the BPOD at the time of data extraction (n = 13). The final cohort (n = 185) represented 77% of those eligible for enrollment. The median age was 6.4 years; 50.8% were male; 25.4% resided in the district of PMH (Southeast); and 88.1% had a histopathologic diagnosis (Table 1). The median follow-up time for all patients was 0.94 years (interquartile range [IQR], 0.25-2.21 years); the median follow-up time was 0.51 years (IQR, 0.15-1.07 years) for deceased patients and 1.72 years (IQR, 0.52-3.22 years) for alive patients.

**Table 1:**
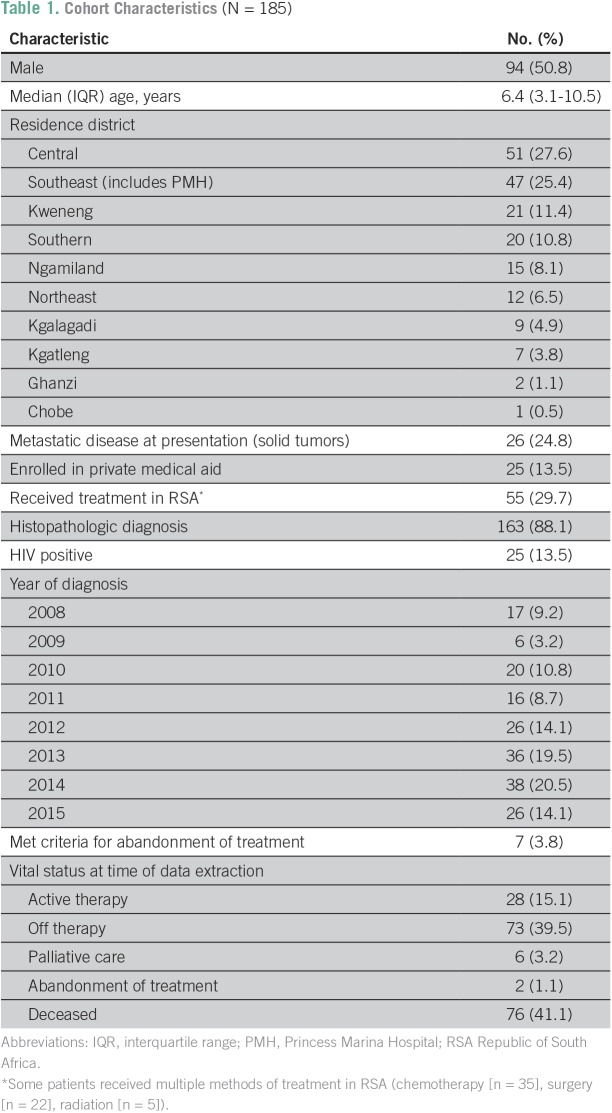
**Cohort Characteristics** (N = 185)

The most common malignancy was leukemia (18.9%), and ALL accounted for 66% of leukemia cases (Table 2). From 2008 to 2011, leukemia accounted for 12% of all malignancies; from 2012 to 2015, leukemia accounted for 22% of all malignancies. Kaposi sarcoma (KS) was the most common malignancy in the 25 children who were HIV-positive (13.5% of 185 children overall) occurring in 16 (64%) of the 25 children who were HIV-positive; all KS diagnoses were associated with HIV. Of the 29.7% of the cohort who received at least a portion of therapy in the Republic of South Africa, 49% had leukemia, and 22% had CNS tumors. Most patients with leukemia (77.1%) or CNS tumors (60%) received some therapy in the Republic of South Africa.

**Table 2:**
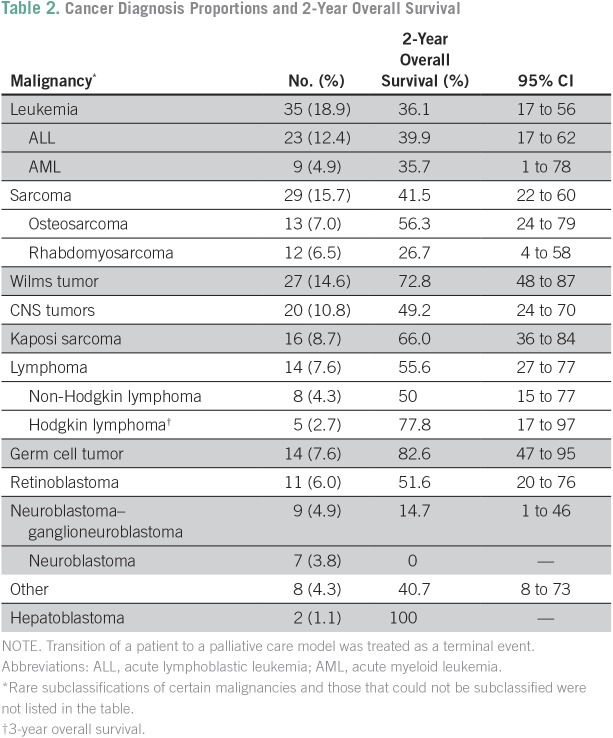
**Cancer Diagnosis Proportions and 2-Year Overall Survival**

**Fig 2:**
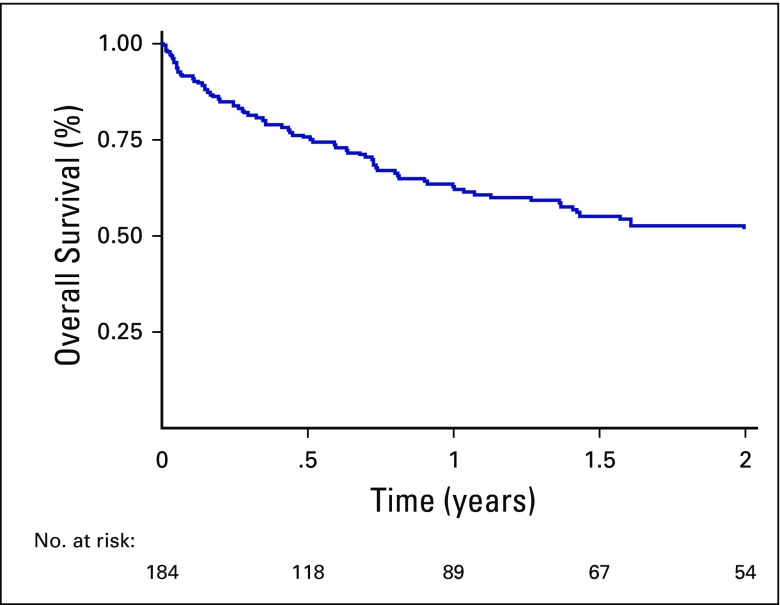
Kaplan-Meier survival curve of 2-year overall survival for all diagnoses.

The majority of the patients who died (60 [71%] of 84 patients overall who died) did so within the first year of diagnosis. The 1-year OS was 64.0% (95% CI, 56% to 71%). The 2-year OS for the study cohort was 52.6% (95% CI, 44% to 60%; Fig 2).

## DISCUSSION

The understanding of pediatric cancer incidence, diagnosis distribution, and treatment outcomes for children in Africa is limited. Historically, noncommunicable diseases like pediatric cancer have not been a focus of health care improvement efforts. Of 18 African countries in which national cancer control plans were reviewed, researchers found that 11 countries, including Botswana, did not include a discussion of pediatric cancer in their plans.^10^ By leveraging the considerable resources and experience of a comprehensive pediatric HIV program in Botswana, PHO services were developed at PMH.^4,11-13^ This initial report of the BPOD demonstrates that pediatric cancer distribution in Botswana appears more similar to reports from the United States or Europe than to other institutional African reports, and that diagnostic and treatment capacity can be developed in a resource-limited setting during a short period of time through collaboration between government and international partnerships.

The incidence of pediatric cancer in the United States (derived from population-level registries) is 16.1 occurrences annually per 100,000 children age 0 to 14 years.^14^ The incidence of pediatric cancer in Botswana and throughout Africa are unclear. National cancer registries in Africa struggle to adequately collect, evaluate, and report on pediatric cancer incidence.^15^ Some countries in Africa do not have population-level cancer registries, and health systems issues create challenges in the optimization of population-level cancer registries that do exist, such as the Botswana National Cancer Registry, although data about pediatric cancers may be limited.^16^ A study in the Republic of South Africa of a population-level registry found that the pediatric cancer incidence was 4.5 cases/100,000 children annually and noted a large racial discrepancy: 3.7/100,000 in the black population versus 11.6/100,000 in the white population. The decreased incidence and racial disparity may have resulted from missed diagnoses and under-reporting among the black population.^17^

Data from the Botswana National Cancer Registry reported in a recent publication described 267 occurrences of pediatric cancer in Botswana in children age 14 years and younger from 2003 to 2008.^15^ Through extrapolation of the US incidence to Botswana, we would expect a minimum of 115 children with cancer per year in those younger than 14 years of age.^5^ However, this extrapolation does not account for the effect of HIV, which increases the risk for certain types of cancer.^18^ In Botswana, 11,000 children are living with HIV.^2^ The number of new pediatric oncology diagnoses per year at PMH appears to under-represent the expected incidence of pediatric cancer in Botswana; there were 185 incident cases of pediatric cancer in this cohort, but only 22 occurrences reported at PMH in the 7 years before establishment of this program. The increase in new incident cases at PMH through the establishment of this program indicates that missed diagnosis is likely a factor in lower-than-expected numbers of occurrences. To improve pediatric cancer recognition and diagnosis in Botswana, more than 350 health care providers at half of the government/mission hospitals were recently trained about presenting signs and symptoms of cancer in children and how to refer these children to PMH.^19^

With the limitations of population-level pediatric cancer registries, the distribution and types of pediatric malignancies in Africa are unclear.^20^ As a hospital-based data base, this BPOD cohort can provide valuable insight on the distribution of pediatric cancers in Botswana, because PMH is the only pediatric cancer center in Botswana. Because most pediatric cancers are more associated with genetics than exposures (as in adult cancers), variation in diagnosis distributions should be more limited across different geographic regions than that seen with adult cancers. The most common malignancies diagnosed in children in the United States include leukemia, 31% (26% of which is ALL); CNS tumors, 21%; neuroblastoma, 7%; non-Hodgkin lymphoma, 6%; and Wilms tumor, 5%.^21^ Institutional reports from African centers typically describe a lower proportion of leukemia, minimal CNS tumors, and a higher proportion of visible tumors like Wilms tumor, Burkitts lymphoma, retinoblastoma, or HIV-associated malignancies (eg, KS).^22-26^ The South African Children’s Tumor Registry more closely resembles the US pediatric cancer distribution pattern with leukemia, lymphomas, renal tumors, and CNS tumors being the most common.^17^ At PMH, leukemia initially represented only 12% of diagnoses but has been the most common diagnosis, at 22%, during the past 4 years of this cohort. Sarcomas, Wilms tumor, and CNS tumors were the next most common, in descending order. Environmental factors—including geography, climate, malaria incidence, and prevalence of viruses such as human herpes virus-8, Epstein-Barr virus, and HIV—play key roles in the distribution of certain pediatric malignancies, particularly with non-Hodgkin lymphoma and KS.^27^ Despite the high prevalence of HIV in Botswana, less than 10% of the total occurrences of pediatric cancer were KS. The evolving cancer distribution pattern in Botswana may be a result of increased awareness of childhood cancer that leads to more children who present for care. The BPOD will complement the Botswana National Cancer Registry by sharing data to ensure that all children with cancer in Botswana are registered. Moreover, improved efforts, both within the health care system and in the community at large, are necessary to create awareness to ensure that all children with cancer in Botswana are recognized and diagnosed promptly.

In many African countries, survival of pediatric cancer either is unknown or contrasts greatly with the 80% survival seen in high-income countries.^21^ In Zambia, in a review of a 2-year cohort of pediatric patients with cancer at the tertiary government hospital, only 8% of patients completed a treatment regimen, and 46% abandoned treatment.^25^ One study of pediatric cancer capacity in 10 LMICs found that the postulated 5-year survival was less than 50% at eight of the sites, including three in Africa.^28^ In this review of a cohort from 2008 to 2015, the 2-year OS rate of all pediatric cancer diagnoses was 52%; however, this could decrease, given that these data are early, because many patients remain at risk for relapse. Unfortunately, the majority of deaths in this cohort occurred within the first year of diagnosis. Research is ongoing to evaluate cause of death of pediatric patients with cancer in Botswana and to identify areas for improvement.

Although many children are achieving a sustained remission from their cancer, significant improvements are necessary to attain treatment outcomes consistent with those seen in high-income countries. Children with Wilms tumor had a 2-year OS rate of 73%, which is comparable to Wilms tumor survival of other African institutional experiences that ranged from 32% to 79%^29-32^ but which lags behind the United States experience of 90%.^21^ Neuroblastoma outcomes were particularly discouraging, with no survivors in this cohort. This result was similar to the experience in Zambia, where no patients with neuroblastoma in a 2-year cohort completed therapy.^25^ A survey of North African centers found that neuroblastoma represented a minor proportion of pediatric malignancies and that outcomes were poor.^33^ Despite treatment for the majority of patients with ALL in South Africa, the 2-year OS rate is 40% compared with nearly 90% in high-income countries.^34^ A higher proportion of unfavorable cytogenetics in patients with ALL may play a role in the poorer-than-expected outcome in these patients; however, additional study is needed.^35^ Efforts continue in Botswana to offer therapy with curative intent regardless of diagnosis or risk stratification and to treat patients with leukemia at PMH. Although pediatric cancer efforts in LMICs often focus on the most common diagnoses with the best prognoses, the goal should be for equitable cancer care for all children with all types of malignancies.

In many pediatric cancer reports from LMICs, treatment abandonment approaches 50%.^25,36,37^ In this cohort, only seven children (3.8%) met criteria for abandonment of treatment, and some returned to care. Although additional study is required, we postulate that many factors influenced the low treatment abandonment rate, including a well-coordinated nationalized health care system, government-sponsored travel between medical facilities, meals provided to inpatients and a caregiver, servicing of a smaller population than other LMICs, efforts of PMH-based social workers, cultural influences, access to a member of the medical team via a shared service phone (7 days a week, 24 hours per day), and the addition of a program nurse care coordinator.^38^

The limitations of the study included its retrospective nature; data were missing particularly for patients from the early stages of the program. Forty-two potentially eligible patients were excluded for a variety of reasons, such as treatment performed outside of PMH or unconfirmed diagnosis of cancer, identity, or outcome. The exclusion of those who were treated at PMH but not enrolled in the BPOD could have led to higher than actual survival outcomes for this cohort.

Though great strides have been made for children with cancer and blood disorders in Botswana since 2007, sustainability will be possible only by developing an infrastructure and human resource capacity to foster local ownership of the program. Over the years, there have been multiple training initiatives, including multiple nurse training programs, which have included multidisciplinary workshops; sponsorship to TXCH for training; integration into the training of medical students, residents, and nursing students; and research mentorship of pediatric residents. In addition to the improvement of the quality of care, these efforts have led to academic scholarship and leadership by health care workers in Botswana and development of local trainers for ongoing initiatives.^8,39-43^ Recently, the partners of this program, through a Memorandum of Agreement with the government of Botswana, committed to build a center of excellence for children’s cancer and blood disorders in Gaborone to provide care for all children with cancer and blood disorders in Botswana, and concurrently, to build local capacity through training and education.^44^ With a significant gift from the Bristol Myers Squibb Foundation, the TXCH Global Hematology-Oncology Pediatric Excellence Program, which includes Botswana, Malawi, and Uganda, will seek to build on this prior experience to move toward the delivery of sustainable and equitable health care to all children in Botswana.^45^
